# Real-Time Monitoring in Home-Based Cardiac Rehabilitation Using Wrist-Worn Heart Rate Devices

**DOI:** 10.3390/s17122892

**Published:** 2017-12-12

**Authors:** Javier Medina Quero, María Rosa Fernández Olmo, María Dolores Peláez Aguilera, Macarena Espinilla Estévez

**Affiliations:** 1 Department of Computer Science, University of Jaen, Campus Las Lagunillas, 23071 Jaén, Spain; mestevez@ujaen.es; 2 Heart Rehabilitation Unit of the Hospital Complex of Jaén, Av. del Ejército Español 10, 23007 Jaén, Spain; mr.fernandez.olmo.sspa@juntadeandalucia.es; 3 Council of Health for the Andalucian Health Service, Av. de la Constitucion 18, 41071 Sevilla, Spain; mdolores.pelaez.sspa@juntadeandalucia.es

**Keywords:** wrist-worn heart rate devices, cardiac rehabilitation, real-time wearable monitoring, fuzzy logic, fuzzy linguistic approach, m-health

## Abstract

Cardiac rehabilitation is a key program which significantly reduces the mortality in at-risk patients with ischemic heart disease; however, there is a lack of accessibility to these programs in health centers. To resolve this issue, home-based programs for cardiac rehabilitation have arisen as a potential solution. In this work, we present an approach based on a new generation of wrist-worn devices which have improved the quality of heart rate sensors and applications. Real-time monitoring of rehabilitation sessions based on high-quality clinical guidelines is embedded in a wearable application. For this, a fuzzy temporal linguistic approach models the clinical protocol. An evaluation based on cases is developed by a cardiac rehabilitation team.

## 1. Introduction

Home-based e-health programs are being increasingly used due to the proliferation of wearable devices and portable medical sensors which are seamlessly integrated into the daily lives of users to monitor vital signs and physical activity [1]. In this way, wearable devices, together with connectivity and ubiquitous computing in mobile applications [2], have provided a solution for monitoring a greater number of patients under prevention and rehabilitation programs in a personalized manner [3].

Moreover, wearable devices have been demonstrated to favor strategies for changes to healthy habits and the promotion of healthy physical activity [4]. To achieve this, a key aspect is to adapt high-quality clinical guidelines and protocols from health centers to home-based solutions [5] in order to provide real-time activity monitoring by means of wearable devices [6].

Motivated by these recent advances, in this work a cardiac rehabilitation program is embedded in a wrist-worn device with a heart rate sensor, which provides real-time monitoring of physical activity during sessions in a safe and effective way. For this, a linguistic approach based on fuzzy logic [7] is proposed in order to model the cardiac rehabilitation protocol and the expert knowledge from the cardiac rehabilitation team. Fuzzy logic has provided successful results in developing intelligent systems from sensor data streams [8,9,10,11,12], and more specifically, it has been described as an effective modeling tool in cardiac rehabilitation [13].

The remainder of the paper is structured as follows: in Section 1.1, the principles and motivation of cardiac rehabilitation together with previous related works are presented; in Section 2, we detail a standardized protocol for cardiac rehabilitation, and based on it, a fuzzy model is proposed for real-time monitoring the heart rate of patients. In Section 3, the developed architecture based on wrist-worn wearable and mobile applications for patients and a cloud web application for the cardiac rehabilitation team is presented; in Section 4, an evaluation of fuzzy modifiers and temporal windows from heart rate sessions is provided by the cardiac rehabilitation team in order to adjust the real-time monitoring of the fuzzy model in practice; and finally, in Section 5, conclusions and suggestions for future works are presented.

### 1.1. Home-Based Cardiac Rehabilitation

Cardiovascular diseases represent a major health problem in developed countries according to the World Health Organization (WHO) [14]. Around 17 million people die annually from cardiovascular pathologies [15]. Fortunately, its prognosis has been improved by primary prevention, drug treatment, secondary prevention, and cardiac rehabilitation, the latter of which has been shown to be the most effective tool [16]. In this way, cardiac rehabilitation has been revealed by multiple studies as effective for reducing morbidity and mortality by around 20–30% in acute myocardial infarctions [17]. *Cardiac rehabilitation* (CR) is defined as the sum of the activities required to favorably influence the underlying cause of heart disease, as well as ensuring the best physical, social and mental conditions, thus enabling patients to occupy by their own means a normal place in society [18]. For these reasons, in recent years, secondary prevention programs and cardiac rehabilitation units (CRUs) have been developed in several countries [17,19,20]. However, there is a lack of accessibility due to several factors, such as lack of time, comorbidities, geographical area, and access to health services [18,20,21]. Minimizing these limitations by means of home-based programs and wearable devices is the motivation of this work, which is underway with respect to the development of CR at the primary and home-care level in order to increase the number of patients who benefit from these programs. These are fundamentally low-risk patients [16].

### 1.2. Related Works

In the literature, we highlighted recent works and reviews where the effectiveness of smart health monitoring systems is described and summarized [15,22,23]. In recent years, some works have been carried out in which information and communication technologies and/or wearable device have enabled the telemonitoring of these patients. These are the most representative of the following works.

In [24], a home-based CR with telemonitoring guidance is evaluated. It includes *individual coaching by telephone weekly* after uploading training data. In [25], a home-based walking training program is presented. The approach includes a health device with four electrodes. At the end of the sessions, the data are transmitted using a mobile device to a monitoring center, which provides indicators of adherence and evaluation. In [26], a combination of e-textiles, wireless sensor networks, and a transmission board provide monitoring of several physiological parameters, such as the electrocardiogram (ECG), heart rate, and body temperature for *future healthcare environments*. In [13], the cardiac and aortic data are collected by wearable t-shirts with embedded electrodes. Then, they are processed by a mobile device to acquire biosignals. In addition, fuzzy logic is presented as an effective modeling tool with monitoring of the vital signs by means of fuzzy rules. In [27], a mobile application uploads the sessions from a wearable device to enable the coaching of health personnel. The wearable device is presented as a data collector without providing a real-time feedback of sessions. In [28], Fitbit wearable sensor devices, and personalized coaching with SMS are proposed. In the same way, an ad hoc application was not embedded in the wearable device. Finally, in [29], the heart rate was measured by the index finger on a built-in camera for one minute at each exercise stage in order to evaluate the quality of the session.

In this way, previous works have foregrounded the relevance and efficacy of integrating cardiac rehabilitation programs (CRPs) into home-based solutions, but with the limitations of non-programmable heart rate sensors or burdensome devices in the early stages of implantation. However recently, a new generation of smart-watches and wrist-worn devices has improved the quality of measures in heart rate (HR), achieving a median error below 5% in laboratory-based activities [30]. Moreover, smart-watches and wrist-worn devices are expected to *be a boon to mHealth technologies* in physical activity sensing thanks to the recent tools and operating systems which enable application development [31].

Based on this context, in this work, we describe real-time monitoring and evaluation of cardiac rehabilitation sessions (CRSs) at home using wearable wrist-worn devices with heart rate sensors. The highlights of this approach are:

- *Wear and play* devices. Wrist-worn devices are noninvasive because of the heart rate sensor is embedded. In addition, they do not require placement of electrodes on the body prior to physical activity. Moreover, they are worn as a watch in an everyday manner.
- *Modeling a theoretical high-quality CRP*. A standardized CRP, which was developed by the CRU of Hospital Complex of Jaen (Spain) was introduced in the home-base approach for each patient’s care in a personalized way. A linguistic approach based on fuzzy logic [7] is included to model the CRP and the expert knowledge from the cardiac rehabilitation team.
- *Real-time smart monitoring* is embedded in the wrist-worn device in order to: (1) show patients the adherence to CRP during physical activity; and (2) prevent unsuitable and inadequate HR ranges.
- *Practical methods* are described for applying the theoretical model to wearable computing.

## 2. Methodology

In Section 2, we detail the standardized protocol for the CRP where this work is focused for proposing a fuzzy model for real-time monitoring of the heart rate of patients by means of wrist-worn wearable devices.

### 2.1. Setting a Cardiac Rehabilitation Program

In this section, we describe a standardized CRP for patients with ischemic heart disease, a disease where patients suffer from a kind of restriction in the blood supply to the tissues. In the literature, several models for handling CRPs have been proposed and analyzed in many countries [32]. In this work, we proposed the use of a general model for cardiac rehabilitation [33] based on the heart rate, which is focused on determining the values of the heart rate training zones in the CRS. This model was developed at the CRU of the Hospital Complex of Jaen, Spain, where this work is centered.

As a previous step before starting the CRP, a first evaluation for each new patient of CR is required in health centers. In this initial evaluation, the patients are connected to an ECG and undergo a controlled cardiac stress test, which is evaluated by a cardiologist in terms of symptoms and blood pressure response for diagnosing patients. From this test, a cardiologist determines the next thresholds for each patient [34]:

- The maximal or peak heart rate ($HR_{max}$), that is, the number of contractions of the heart per minute (bpm) when it is working at its maximum capacity without severe problems.
- The basal or resting heart rate ($HR_{rest}$), that is, the bpm when the patient is awake, relaxed, and has not recently exercised.
- The first ventilatory threshold ($VT_{1}$), that is, the bpm which represents a level of intensity when blood lactate accumulates faster than it can be cleared, this being related to the aerobic threshold.
- The second ventilatory threshold ($VT_{2}$), that is, the bpm which represents the point where lactate is rapidly increasing with an intensity that generates hyperventilation; this being related to the anaerobic threshold.

Once patient thresholds are defined in the health center, a set of sessions are designed for configuring CRP by the cardiac rehabilitation team defining the:

- Duration range. The exercise duration of sessions, which is increased from initial sessions in an interval of (15–20) min to an interval of (30–40) min for trained patients [35].
- The optimal heart rate training zones (OHRTZs). These are defined by the clinical protocol in each session, as percentage ranges $\left[p_{+}^{*},p_{-}^{*}\right]$ from $HR_{max}$ and $HR_{rest}$. The methodology of Marvonen [33] allows translating the percentage range to absolute *bpm* $\left[r_{+}^{*},r_{-}^{*}\right]$ that is defined by $r^{*}=HR_{rest}+p^{*}\dot{(}HR_{max}-HR_{rest})$.
  The middle point between $\left[r_{+}^{*},r_{-}^{*}\right]$ is known as *target heart rate* $HR_{tar}$, which is related to the ideal heart rate to maintain in the session.
- Duration of the progressive stage ($d_{w}$). The progression of HR within from basal state needs for a lineal increase, which starts from the resting point until reaching the OHRTZ. The duration of this progressive stage is defined in minutes.

### 2.2. A Fuzzy Model for Real-Time Monitoring and Evaluation of Cardiac Rehabilitation Sessions

In this section, we describe a fuzzy model for real-time monitoring and evaluation of the heart rate of patients in accordance with the cardiac rehabilitation program described in Section 2.1.

This fuzzy model is proposed to describe, in real-time, the heart rate stream, which is composed of the measured values and the time-stamps when they are collected by the heart rate sensor. In Section 2.2.1, we focus on fuzzification of the measures from heart rate sensor. In the Section 2.2.3, we describe a fuzzy aggregation of the terms using temporal windows. Moreover, in order to model the progressive stage, a fuzzy transformation from progressive to maintenance stage is detailed in Section 2.2.2. Finally, in Section 2.2.4, we detail an interpretable evaluation based on the previous steps to describe the further heart rate stream at the end of the rehabilitation session.

#### 2.2.1. Fuzzification of Heart Rate Measures by Optimal Heart Rate Training Zones

In this section, we describe a linguistic approach based on fuzzy logic for the OHRTZ. In fuzzy logic methodology, a variable can be defined by means of terms, which are described by means fuzzy sets. Each fuzzy set is defined in terms of a membership function which is a mapping from the universal set to a membership degree between 0 and 1.

Based on the fuzzy logic methodology, we proceed to describe the HR under a linguistic representation defined by the parameters from the CRP, detailed in Section 2.1. Specifically, we propose three intuitive terms {*low, adequate and high*}, which are defined by fuzzy sets, for describing the variable *heart rate*, which is measured by a 2-tuple value $\bar{hr_{i}}=hr_{i},t_{i}$. $hr_{i}$ represents a given value in the heart rate stream and $t_{i}$ its time-stamp. Hence, the heart rate stream is composed of a set of measured values $S_{\bar{hr}}=\left\{\bar{hr_{0}},\ldots ,\bar{hr_{i}},\ldots ,\bar{hr_{n}}\right\}$ which are collected by the heart rate sensor.

In this section, we focus on the fuzzification of a heart rate measure individually, $\bar{hr_{i}}$. On one hand, because of prior definitions in cardiac rehabilitation, which are, (1) the OHRTZs as values of HR between the ranges $\left[r_{+},r_{-}\right]$; and (2) the ventilatory thresholds $\left[VT_{1},VT_{2}\right]$ as the efficient and safe ranges of aerobic physical activity, we define the term as *adequate*. This term is described by a fuzzy set characterized by a membership function whose shape corresponds to a trapezoidal function. The well-known trapezoidal membership functions are defined by a lower limit $l_{1}$, an upper limit $l_{4}$, a lower support limit $l_{2}$, and an upper support limit $l_{3}$ (See Equation (1)):

(1) $$TS\left(x\right)\left[l_{1},l_{2},l_{3},l_{4}\right]=\left\{\begin{array}{cc} 0 & x\leq 0\\ \left(x-l_{1}\right)/\left(l_{2}-l_{1}\right) & l_{1}\leq x\leq l_{2}\\ 1 & l_{2}\leq x\leq l_{3}\\ \left(l_{4}-x\right)/\left(l_{4}-l_{3}\right) & l_{3}\leq x\leq l_{4}\\ 0 & l_{4}\leq x\; \end{array}\right.$$

For the term *adequate*, the fuzzy set is characterized by the trapezoidal membership function that is defined by Equation (2):

(2) $$\mu_{adequate}\left(hr_{i}\right)=TS\left(hr_{i}\right)\left[VT_{1},r_{-}^{*},r_{+}^{*},VT_{2}\right],VT_{1}<r_{-}^{*}<r_{+}^{*}<VT_{2}$$

On the other hand, with $VT_{2}$ being the threshold from aerobic to anaerobic activity, and $r_{+}^{*}$ the upper limit range for OHRTZs, we define the term *high*, which is described by a fuzzy set characterized by the trapezoidal membership function that is defined by Equation (3):

(3) $$\mu_{high}\left(hr_{i}\right)=TS\left(hr_{i}\right)\left[r_{+}^{*},VT_{2},VT_{2},VT_{2}\right],VT_{2}>r_{+}^{*}$$

In a similar way, with $VT_{1}$ being the lower threshold of the aerobic activity and $r_{-}$ the lower limit range for OHRTZs, we define the term *low*, which is described by a fuzzy set characterized by the trapezoidal membership function that is defined by Equation (4):

(4) $$\mu_{low}\left(hr_{i}\right)=TS\left(hr_{i}\right)\left[VT_{1},VT_{1},VT_{1},r_{-}^{*}\right],VT_{1}<r_{-}^{*}$$

**Figure 1:**
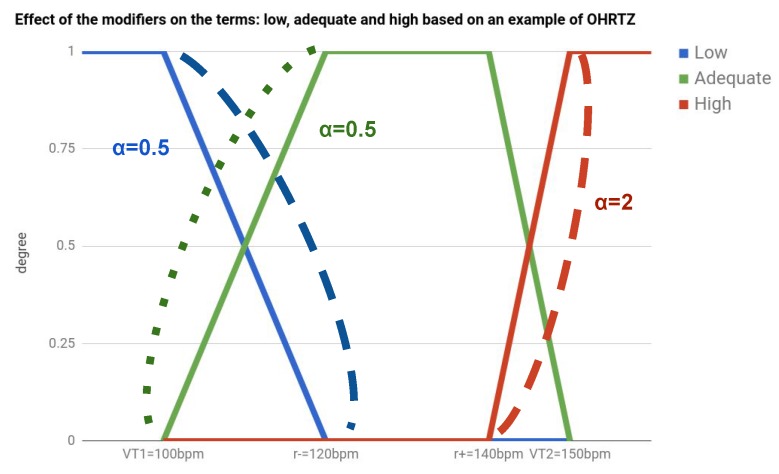
Example of membership functions for the terms low, normal, and high. In the example, the optimal heart rate training zones (OHRTZs) of the sessions are for trained patients, which are closer to $VT_{2}$ than to $VT_{1}$. In the example of modifiers, the impacts of the weak modifier in short-dashed lines and the strong modifier in long-dashed lines are shown.

The relation between the thresholds from cardiac rehabilitation program and the membership functions is shown in Figure 1. Moreover, thanks to the use of linguistic modifiers, in fuzzy logic, we can model different semantics over the membership functions for describing the linguistic terms [36]. To represent the impact of a linguistic modifier *m* over a linguistic term *v*, such as *great* or *fair*, a straightforward power operation of the membership function is proposed [37] and defined by $\mu_{m,v}\left(x\right)=\mu_{v}{\left(x\right)}^{\alpha_{m}}$.

If $\alpha_{m}<0$, we obtain a weak modifier, such as *fair*; and a strong modifier with $\alpha_{m}>0$, such as *great*. In Figure 1, we describe the impact of the linguistic modifiers, and in Section 4, we describe the comparative results of provided by the cardiac rehabilitation team.

At this point, based on the current value of the heart rate $hr_{i}$ and the thresholds for the session $\left[VT_{1},VT_{2}\right]$ and $\left[r_{+}^{*},r_{-}^{*}\right]$, we are able to calculate the degree of the fuzzy terms {*low, adequate, and high*} in order to advise the patient in real-time with respect to the adequacy of the sessions.

The degrees of membership of the HR to the fuzzy sets {*low, adequate, and high*} can provide an intuitive evaluation for the real-time monitoring of sessions in wrist-worn wearable devices. For example, in this work, gradually changing colors in the evaluation of the HR are used to paint the screen of the wearable device and to evaluate the session using a 4-star scale, as described in Section 3.

However, in practice, it is necessary to handle additional issues in order to provide real-time monitoring during the rehabilitation sessions: the monitoring in the progressive stage and the temporal evaluation of heart rate streams.

#### 2.2.2. Fuzzy Transformation from the Progressive to Maintenance Stage

In the literature there is a lack of proposals for modeling of the progressive stage in cardiac rehabilitation. This is related to the fact that it does not contain critical HRs. To resolve this issue, we propose a straightforward method to translate the model of OHRTZs from the aerobic state to define the initial basal state. In this way, the basal state is described by the following parameters:

- Basal ranges $\left[r_{+}^{0},r_{-}^{0}\right]$ in bmp, where the HRs of patient are adequate in order to start the session.
- Lower basal threshold in bmp $VT_{1}^{0}$. This represents a minimal value of HR not recommended before starting the session.
- Upper basal threshold in bmp $VT_{2}^{0}$. This represents a maximal value of HR not recommended before starting the session.

Next, for calculating the time evolution in real-time within the progressive stage, we define a weight progression $w=\Delta t_{0}/d_{w},w\in \left[0,1\right]$, where $\Delta t_{0}$ is the duration of session in the current time $t_{0}$ and $d_{w}$ is the total duration of the progressive stage defined by the cardiac rehabilitation team.

Based on the temporal evolution of the weight progression as well as the initial and final values of each threshold, we can define the threshold in the progressive stage for each current time frame using a linear progression as shown in Equation (5).

(5) $$\begin{array}{c} r_{-}\left(w\right)=r_{-}^{0}+\left(r_{-}^{*}-r_{-}^{0}\right)\;\cdot \;w,w=\Delta t_{0}/d_{w}\\ r_{+}\left(w\right)=r_{+}^{0}+\left(r_{+}^{*}-r_{+}^{0}\right)\;\cdot \;w,w=\Delta t_{0}/d_{w}\\ VT_{1}\left(w\right)=VT_{1}^{0}+\left(VT_{1}-VT_{1}^{0}\right)\;\cdot \;w,w=\Delta t_{0}/d_{w}\\ VT_{2}\left(w\right)=VT_{2}^{0}+\left(VT_{2}-VT_{2}^{0}\right)\;\cdot \;w,w=\Delta t_{0}/d_{w} \end{array}$$

**Figure 2:**
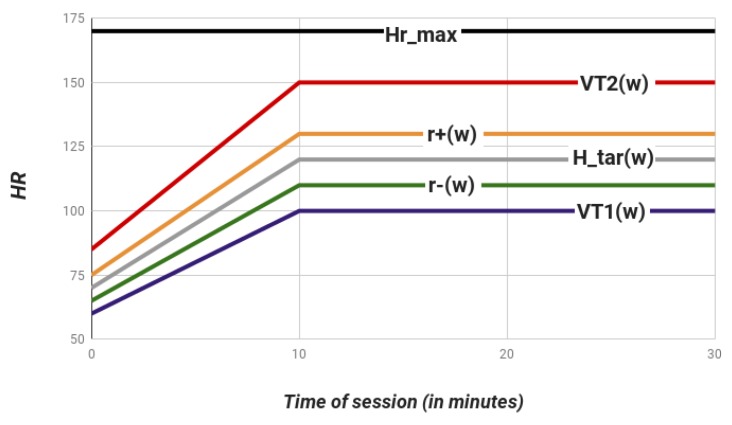
Evolution of the values of parameters from progressive to maintenance stages for a rehabilitation session: duration range (30 min), duration of progressive stage (10 m) , OHRTZ $r_{+,-}^{*}=$ [130 bpm, 110 bpm], $HR_{max}=170$ bpm, and $VT_{1,2}=$ [100 bpm, 150 bpm]. This includes the basal ranges $[r_{+}^{0},r_{-}^{0}]=$ [65 bpm, 75 bpm], and the lower and upper basal threshold $VT_{1,2}^{0}=$ [60 bpm, 85 bpm] for the patient. HR: heart rate; bpm: number of contractions of the heart per minute.

In Figure 2, we show an example of a CRS, where the linear progression of thresholds from progressive to maintenance stages is plotted.

#### 2.2.3. Fuzzy Temporal Aggregation of the Heart Rate Stream

In the practice of developing based-sensor systems, the temporal component in the data streams is a critical aspect to analyze [38]. For example, in a given current time when we evaluate the heart rate sensor stream, we can take into account the last single sample of HR or calculate an average within a sliding window.

In this work, we propose fuzzy temporal aggregation [8], which provides a model to: (1) weight linguistic terms based on temporal membership functions; (2) define progressive and interpretable temporal linguistic terms; and (3) give flexibility in the presence of eventual signal loss or variance in the sample rate.

Based on previous works [8,39], we have integrated a fuzzy aggregation of the terms in the heart rate sensor stream using fuzzy temporal windows, which are straightforwardly described in function of the distance from each sample time-stamp $ts=\{t_{0},\ldots ,t_{n}\}$ to the current time $\Delta t_{i}=t_{i}-t_{0}$.

First, the degrees of a fuzzy term, in our case V={low,adequate,high}, are weighted by the degree of their time-stamps evaluated by a fuzzy temporal window $T_{k}$ defined by Equation (6).

(6) $$V_{r}\cap T_{k}\left(\bar{hr_{i}}\right)=V_{r}\left(hr_{i}\right)\cap T_{k}\left(\Delta t_{i}\right)\in \left[0,1\right]$$

Secondly, the degrees of membership over the fuzzy temporal window are aggregated using the t-conorm operator in order to obtain a single degree of both fuzzy sets $V_{r}\cap T_{k}$ by Equation (7).

(7) $$V_{r}\cup T_{k}\left(S_{\bar{hr}}\right)=\underset{\bar{hr_{i}}\in S_{\bar{hr}}}{⋃}V_{r}\cap T_{k}\left(\bar{hr_{i}}\right)\in \left[0,1\right]$$

We note several fuzzy operators can be applied to implement the aggregation. However in this paper, we propose a fuzzy weighted average [40] as is recommended in the case of high sample rates from wearable sensors [8]. The aggregation process is defined by Equation (8).

(8) $$\begin{array}{c} V_{r}\cup T_{k}\left(S_{\bar{hr}}\right)=\frac{1}{\sum T_{k}\left(\Delta t_{i}\right)}\sum_{}V_{r}\left(hr_{i}\right)\times T_{k}\left(\Delta t_{i}^{j}\right)\in \left[0,1\right] \end{array}$$

The definition and adequacy of several temporal windows, which model the evolution of linguistic terms in the heart rate stream, are discussed in Section 4.

#### 2.2.4. Evaluating the Cardiac Rehabilitation Sessions

Previous sections describe real-time monitoring of heart rate stream within a wearable device based on fuzzy logic. Once the rehabilitation session has been finished by the patient, evaluating the further session at the end to provide a feedback is fairly intuitive.

Based on the degree of a fuzzy term $V_{r}$ and its temporal window $T_{r}$, we compute an accumulative degree in the complete data stream $S_{\bar{hr}}$ by Equation (9).

(9) $$\begin{array}{c} low\left(S_{\bar{hr}}\right)=\sum low\cup T_{low}\left(\bar{hr_{i}}\right)\\ adequate\left(S_{\bar{hr}}\right)=\sum adequate\cup T_{adequate}\left(\bar{hr_{i}}\right)\\ high\left(S_{\bar{hr}}\right)=\sum high\cup T_{high}\left(\bar{hr_{i}}\right) \end{array}$$

Under this approach, the accumulative degrees are calculated as the average degree of the terms in the heart rate stream ahead, providing upright and interpretable analytical data for the session. For example, the accumulative value of the term *adequate* has been used to fill a 4-star scale in the mobile application of this work in order to provide an evaluation of the rehabilitation session for the patient.

## 3. Development as a Wearable Mobile Cloud Platform

In this section, we describe the technical development of the proposed approach to be deployed in wearable wrist-worn devices, mobile devices, and a cloud web platform. The proposed architecture is inspired by current advances in wearable and mobile development tools [41], which provide real-time monitoring in wearable devices and data synchronization between mobile and web applications.

**Figure 3:**
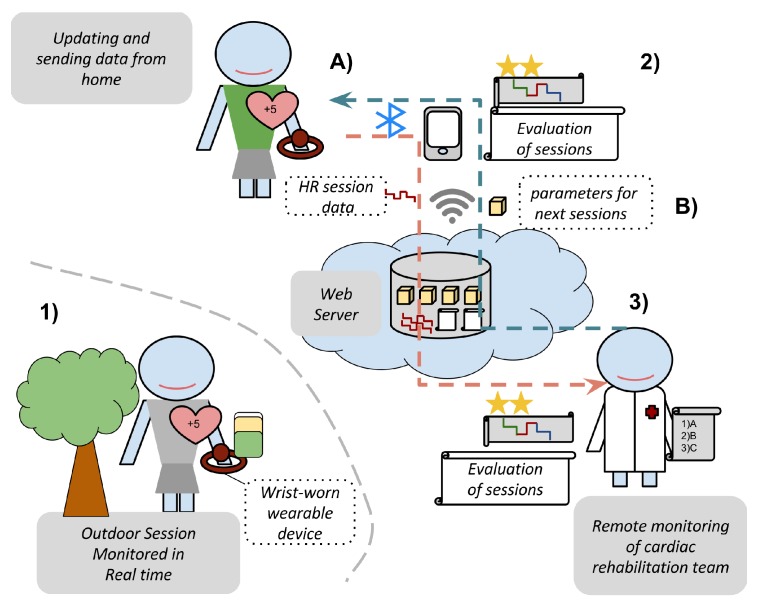
Architecture of components: (**1**) wearable device with real-time monitoring; (**2**) mobile application for evaluating the sessions; and (**3**) a web platform for evaluating the sessions by the cardiac rehabilitation team. The data from the patient and the team are synchronized (**A**) from wearable to mobile by Bluetooth; and (**B**) from mobile to cloud services by 4G/WiFi.

For the client, we have implemented two applications using Android Platform [42], both in wearable wrist-born and mobile devices. On the server side, we have implemented a web server under Java Tomcat, which web services orchestrate and synchronize the flow data between the cardiac rehabilitation team and patients. In Figure 3, we show the architecture and data flow of components.

Hence, the approach includes three applications: a wearable application, a mobile application, and a web application, whose use cases are:

- A cloud web application for the cardiac rehabilitation team. Requiring previous authentication through login credentials, this includes the next use cases:
  - Registration and updating of patient data, including the thresholds for CRP: maximal heart rate $HR_{max}$, ventilatory thresholds $\left[VT_{1},VT_{2}\right]$, basal ranges $\left[r_{+}^{0},r_{-}^{0}\right]$ and basal limits $\left[VT_{1}^{0},VT_{2}^{0}\right]$.
  - Creation and modification of the parameters of the sessions in the rehabilitation programs. They are: optimal heart rate training zones $\left[r_{+}^{*},r_{-}^{*}\right]$, duration range $\left[d_{1},d_{2}\right]$, and duration of progressive stage $d_{w}$.
  - Showing the CRSs from patients. The sessions developed by patients are synchronized from the mobile application to the web server. From them, the cardiac rehabilitation team can observe: (1) the raw data from the HR of the session in a timeline (with an option to zoom and scale); (2) the real-time monitoring provided for the patient using gradual colors: *blue, green, red* based on the degree of the terms {low,adequate,high}, respectively; and (3) a summarized indicator which evaluates the session using a 4-star scale.
- A mobile application for patients in order to show the sessions and communicate the data between the web server and the wearable devices. It has been developed for Android and included the next use cases:
  - Synchronization of the parameters of the next sessions from the CRP, which are defined by the cardiac rehabilitation team and are collected in the web server, in the mobile device using a web service under wireless network technology (3G/4G or WiFi).
  - Synchronization of the session data from the wrist-worn wearable device into the mobile device using an ad-hoc Bluetooth connection.
  - Uploading of the session data from the mobile device into the web server using a web service under wireless network technology (3G/4G or WiFi).
  - Showing and evaluating the CRSs. In the same way as the cardiac rehabilitation team, patients can observe the following information in their mobile devices for each session: (1) the raw data from the HR of sessions in a timeline; (2) the real-time monitoring provided using gradually changing colors *blue, green, red*; and (3) a summarized indicator of the session using a 4-star scale.
- A wearable application for patient in order to develop the sessions with regard to the CRP. This has been developed for Android to include the next use cases:
  - Updating the the parameters of sessions from the mobile device into the wrist-worn wearable device using an ad-hoc Bluetooth connection.
  - Monitoring the session in the wearable device with regard to the CRP providing a real-time monitoring by means of showing (1) the current HR of the patient; (2) the target HR; (3) a graphical evaluation using gradually changing colors *blue, green, red* based on the degree of the terms {low,adequate,high} respectively; and (4) the time of session and the graphical time progression in proportion to the proposed duration.
  - To synchronize the data of sessions, which contains the monitoring and raw HR, from the wrist-worn wearable device into the mobile device using an ad hoc Bluetooth connection.

**Figure 4:**
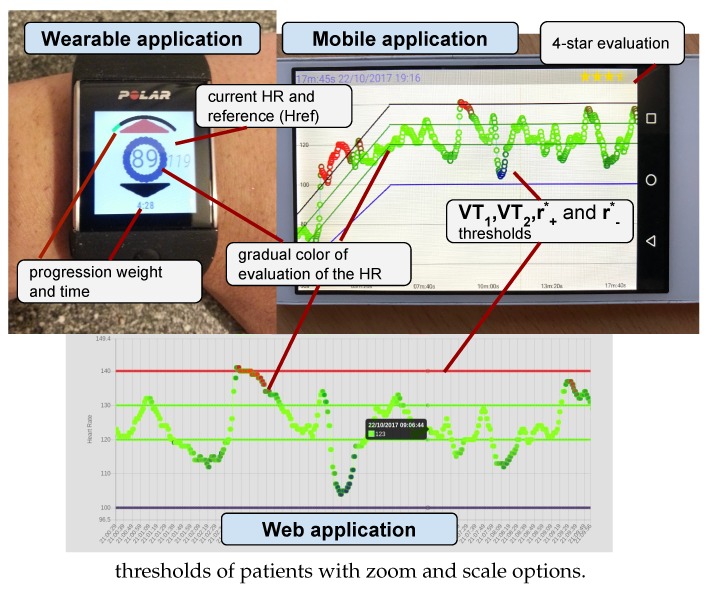
Pictures of the wearable, mobile, and web applications. In the wearable application in Polar M600, we show (1) a gradual color change in the evaluation of the HR; (2) progression and total time; and (3) the current and reference HR. In the mobile application, the $VT_{1}$, $VT_{2}$, $r_{+}^{*}$ and $r_{-}^{*}$ thresholds and the 4-star evaluations are described. In the web application, the cardiac rehabilitation team has access to heart streams and $VT_{1}$, $VT_{2}$, $r_{+}^{*}$ and $r_{-}^{*}$ thresholds of patients with zoom and scale options.

Images from wearable, mobile, and web applications are shown in Figure 4, where we detail the evaluation and real-time monitoring developed under the methodology described in Section 2 by means of the technological components of the approach.

Polar M600 (https://www.polar.com/us-en/products/sport/M600-GPS-smartwatch) was chosen as an Android Wear device due to the high-quality optical heart rate monitor. The strength specifications of Polar M600 include : (1) optical heart rate measurement with six LEDs; (2) its waterproof nature (IPX8 10 m); (3) low weight (63 g); (4) reduced dimensions (45 × 36 × 13 mm); and (5) long-life battery (500 mAh Li-pol for a 2-day average uptime per charge or 8 h of training).

Based on the further evaluation of [43], Polar M600 is highly accurate. The HR value is ±5 bpm or less from the ECG HR value during periods of steady-state sports (cycling, walking, jogging, and running), which are the focus of cardiac rehabilitation. However, the accuracy was reduced during some intensity change exercises. No statistically significant was found in this sample on the basis of sex, body mass index, VO2max, skin type, or wrist size.

## 4. Results

In this section, we present an evaluation of the fuzzy model for real-time monitoring of cardiac rehabilitation sessions (CRSs) at home using wearable wrist-worn devices with heart rate sensors. As we discussed previously in Section 2, the theoretical aspects of cardiac rehabilitation are well defined in the literature. However, we can model different semantics over the membership functions for describing the linguistic terms by means of linguistic modifiers and temporal windows. In the next sections, we discuss the impact and adequacy of them based on the expert knowledge of a cardiac rehabilitation team.

### 4.1. Impact of Modifiers over Linguistic Terms

In this section, we describe an evaluation of the linguistic modifiers for the terms defined in Section 2.2.1. As we detailed previously, the OHRTZs define the ranges where the values of HR are totally *adequate*, $VT_{1}$ defines the basal-aerobic threshold from which inferior values of HR are totally *low*, and $VT_{2}$ the aerobic-anaerobic threshold from which upper values of HR are totally *high*. However, the values of HR between these optimal zones need to change gradually. This progression between optimal zones has been modeled and evaluated using several modifiers.

In this work, we have evaluated three models using different modifiers to adjust the progression in the trapezoidal membership functions of the terms *low, adequate, and high* with regards to Section 2.2.1.

**Figure 5:**
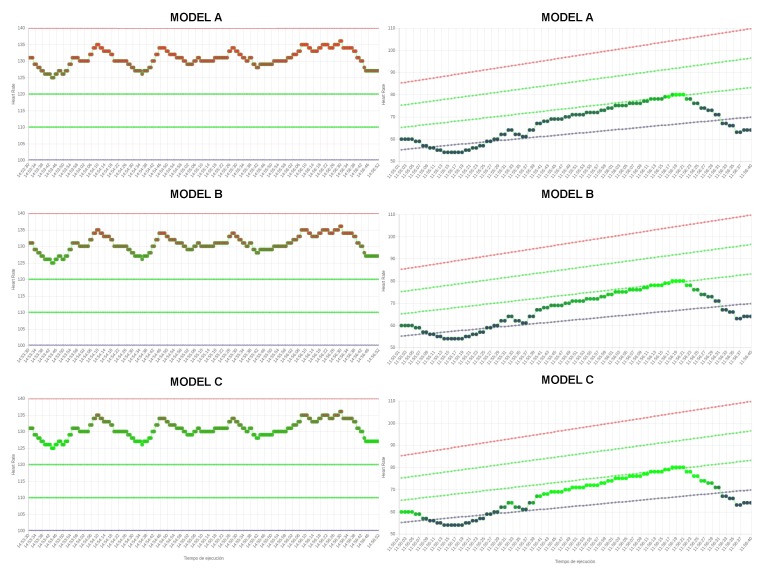
Impact of the fuzzy modifiers on heart rate streams. Heart rate is plotted using gradually changing colors *blue, green, red* based on the degree of the terms {low,adequate,high}, respectively. Green dotted lines determine the OHRTZs of patient. Blue and red dotted lines determine aerobic thresholds $VT_{1},VT_{2}$ of the patient, respectively. The impact of the models A, B and C for a case of high–adequate HRs (**right**); and the impact of the models A, B, and C for a case of low–adequate HRs (**left**).

First, we have evaluated low–adequate values of the heart rate by means of three models: (A) a severe model, where the *low* term is strong and the *adequate* term is weak; (B) a neutral model, where neutral modifiers are applied to both terms; and (C) a yielding model, where the *adequate* term is stronger than the *low* term, which is weaker. The strong, neutral, and weak properties have been defined by the parameters α=0.5,α=1, and α=2.0 of the modifier, respectively. In Figure 5, we show a representation of the impact of the modifiers on the degree of the linguistic terms in a HR stream.

To evaluate the impact of fuzzy modifiers, we have included a survey of 10 cases with key fragments of low values from the heart rate of a sessions, which were colored with blue and green, based on the degree of the terms low and adequate, respectively. In Figure 5, we show an example of a survey case. In a clinical session, the cardiac rehabilitation team evaluates them using a 5-point Likert scale: *{value -2, value -1, value 0, value +1, value +2}*, for which results are detailed in Table 1.

Second, in a similar way, high–adequate values of heart rate have been evaluated by means of three models: (A) a severe model; (B) a neutral model; and (C) a yielding model. A second survey, which contains 10 cases with key fragments of high values from the heart rate of sessions, was evaluated by the cardiac rehabilitation team using the 5-point Likert scale. Results are detailed in Table 1 and two examples of cases from the surveys are presented in Figure 5.

### 4.2. Impact of Temporal Window over Linguistic Terms

In this section, we describe an evaluation on the fuzzy temporal windows over linguistic terms which describe the heart rate stream during rehabilitation sessions. As we detailed previously, the theoretical thresholds of the heart range zones from CRP are defined theoretically missing the temporal permanence in OHRTZs. In some critical situations when patients develop the CRP, the evolution of heart rate between OHRTZs is prompt and inconstant. In those cases, the adherence and adequacy could not be just defined by the current value of HR.

In order to analyze the impact of the temporal windows, a survey with 15 key fragments of prompt and inconstant heart rate streams from the CRSs was designed to evaluate three temporal windows. The cardiac rehabilitation team from the Hospital Complex of Jaen (Spain) analyzed the impact of temporal windows for each term {low,adequate,high} based on their expert knowledge.

**Figure 6:**
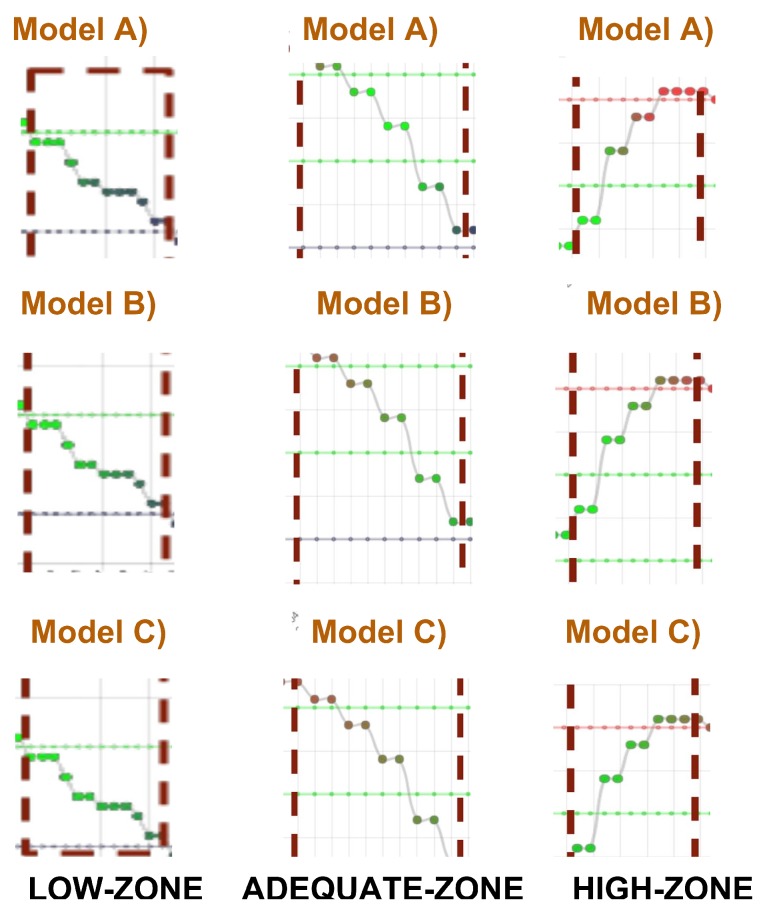
Impact of temporal windows on a case of prompt heart rate streams within the zones described by the linguistic terms {low,adequate,high}. Based on expert evaluation: Model A (short-term temporal window) suits in *high* zones detecting immediately critical HRs; Model B (middle-term temporal window) suits in *adequate* zone requiring a minimal permanence within; and Model B suits in the *low* zone without critical differences with regard to other models.

The temporal windows to evaluate are ($t_{1}$): the last single sample; ($t_{2}$): a 3–5 s window; and ($t_{3}$): a 5–10 s window. In the two last cases ($t_{2}$ and $t_{3}$), we have defined the next fuzzy temporal windows $\mu_{t2}\left(\Delta t_{i}\right)=TS\left(3s,3s,3s,5s\right)$ and $\mu_{t3}\left(\Delta t_{i}\right)=TS\left(5s,5s,5s,10s\right)$ based on the temporal fuzzification described in Section 2.2.3. In Figure 6, we detail an example of the semantics and impact of the three temporal windows on a heart rate streams.

In Table 2, we show the results of the evaluation described by a 5-point Likert scale: *{value −2, value −1, value 0, value +1, value +2}*. We can observe that the short-term temporal window $t_{1}$ is more recommendable when evaluating the term *high* because of corresponding critical values of HR, which require an immediate response from the patient to decrease the heart rate, whereas the long-term window $t_{3}$ is strongly not recommended. On the other hand, the longer temporal window $t_{2}$ is more properly related to the temporal term *adequate* because of the correct adherence the HR stream needs for a temporary stabilization in OHRZs. Finally, the term *low* is more appropriate with the temporal window $t_{2}$, and is adequate with other windows too.

### 4.3. Discussion

On one first hand, from the results presented in Section 4.1, where the impact of modifiers are evaluated, we observe the preferences for the neutral model, where neutral modifier $\alpha_{m}=1.0$ is applied to the linguistic terms. It indicates the non-predominance of a linguistic term over another when defining transition zones between OHRTZs and the aerobic thresholds $VT_{1},VT_{2}$. We note that in the case of high values of HR, which are more sensitive for patients, the yielding model is strongly not recommended by the experts.

On the other hand, based on expert evaluation presented in Section 4.2 where the impact of temporal windows are evaluated, we note that the short-term temporal window suits in *high* zones detecting immediately critical heart rates. Model B (the middle-term temporal window) suits in the *adequate* zone requiring a minimal permanence within, and also suits in the *low* zone without critical differences with regard to other models.

In this way, we can note the adequacy of the clinical protocol for real-time monitoring the CRSs in wrist-worn devices. In addition, the use of a fuzzy model including modifiers and temporal windows has provided a methodology to obtain more accurate terms. This methodology can be extended to model other health contexts based on data stream processing.

Although previous works have been mainly focused on ECG sensors [13,26], the use of a wrist-worn device with the new generation of heart rate sensors provides high accuracy with respect to ECGs [43] for low-risk patients performing low- and medium-intensity exercise. The proposed approach has been implemented within Polar M6000 with Android Wear. Moreover, this wrist-worn device is noninvasive, light and comfortable, but with powerful computing capacity.

On translating the approach to other devices and health contexts, we advise that the quality and precision of heart rate is critical to ensure patient safety. High-risk patients and those with other pathologies could require more accurate devices such as ECGs. In this way, the proposed wrist-worn device just provides a measurement of HR. ECG devices could provide further signal processing of HR where heart rate variability or QRS could be described by means of the here-proposed linguistic terms and fuzzy temporal windows, due to the expanding importance of short-term beat windows in patient analysis [44].

Finally, we note the light processing to compute our methodology, which is based on fuzzy logic, enabling low-cost wrist-worn devices to incorporate it without a computational burden. Other approaches based on similar devices, such as the Fitbit [28] or Garmin [45], could be extended to develop embedded applications providing real-time monitoring during rehabilitation sessions.

## 5. Conclusions and Future Work

The main motivation of this work is enabling the high-quality real-time monitoring of CRPs at the homes of patients, designed and supervised remotely by the cardiac rehabilitation team. For this, we have proposed: (1) integrating a high-quality protocol based on clinical guidelines for monitoring the HR of the patient in a personalized way; (2) providing a real-time monitoring during sessions using a wearable wrist-worn device with heart rate sensor; and (3) using a wearable-mobile-cloud platform for collecting and synchronizing data between patients and the cardiac rehabilitation team.

The methodology of this work has been focused on modeling the theoretical approaches for developing an wearable application for real-time monitoring using wrist-worn devices. In order to address this challenge, first, a fuzzy model is proposed to describe under a linguistic approach the heart rate stream by means of three representative terms: *low, adequate, and high*. Fuzzy modifiers and fuzzy temporal windows are included in the methodology. The fuzzy approach provides a flexible evaluation of the HR stream: (1) enabling a intuitive real-time monitoring in wrist-worn wearable devices during sessions; and (2) providing visual and gradual advice, for which intensity is related to the degree of the terms.

On the other hand, an evaluation of fuzzy modifiers and fuzzy temporal windows is included to generate more accurate and flexible terms. In Section 4, the impact of fuzzy modifiers and temporal windows over the linguistic terms is analyzed by means of surveys based on cases. They have been evaluated by the cardiac rehabilitation team from the Hospital Complex of Jaen (Spain), indicating the most appropriated semantics for each linguistic term.

In future works, the approach will be extended to generate linguistic recommendations and summaries of the further sessions for the patients and the cardiac rehabilitation team. In this work, we have introduced the aggregation as a straight indicator in a 4-star scale, but a further analysis of the heart rate stream will provide intelligent and automatic feedback for cardiologists to detect weak points in the sessions of patients.

## Figures and Tables

**Table 1:** The columns are related in order of: (1) survey; (2) model; (3) modifiers; and then percentages of responses for (4) value −2; (5) value −1; (6) value 0; (7) value +1; and (8) value +2.

Survey	Model	α Modifiers	*Value* −2	*Value* −1	*Value* 0	*Value* +1	*Value* +2
**High values**	**severe**	$\alpha_{high}=2,\alpha_{adequate}=0.5$	0	0.2	0.6	0.2	0
	**neutral**	$\alpha_{high}=\alpha_{adequate}=1$	0	0	0.2	0.5	0.3
	**yielding**	$\alpha_{high}=0.5,\alpha_{adequate}=2$	0.3	0.4	0.3	0.0	0
**Low values**	**severe**	$\alpha_{low}=2,\alpha_{adequate}=0.5$	0	0.3	0.3	0.3	0.1
	**neutral**	$\alpha_{low}=\alpha_{adequate}=1$	0	0	0.2	0.4	0.4
	**yielding**	$\alpha_{low}=0.5,\alpha_{adequate}=2$	0	0.4	0.5	0.1	0

**Table 2:** The columns are related in order of: (1) term; (2) model; and the percentage of responses for: (3) value −2; (4) value −1; (5) value 0; (6) value +1; and (7) value +2.

Term	Model	*Value* −2	*Value* −1	*Value* 0	*Value* +1	*Value* +2
**High**	$t_{1}$	0	0	0.3	0.3	0.4
	$t_{2}$	0	0	0.3	0.4	0.3
	$t_{3}$	0.3	0.4	0.3	0	0
**Adequate**	$t_{1}$	0	0	0.3	0.4	0.3
	$t_{2}$	0	0	0.2	0.2	0.6
	$t_{3}$	0	0.2	0.4	0.4	0
**Low**	$t_{1}$	0	0	0.3	0.4	3.0
	$t_{2}$	0	0	0.3	0.3	0.4
	$t_{3}$	0	0	0.3	0.4	3.0

## Abbreviations

The following abbreviations are used in this manuscript:

CR	Cardiac Rehabilitation
CRU	Cardiac Rehabilitation Units
CRP	Cardiac Rehabilitation Program
CRS	Cardiac Rehabilitation Sessions
ECG	Electrocardiogram
VT1	First Ventilatory Threshold
VT2	Second Ventilatory Threshold
HR	Heart Rate
bpm	Number of contractions of the heart per minute
OHRTZ	Optimal Heart Rate Training Zone
